# Developing Mood-Based Computer-Tailored Health Communication for Smoking Cessation: Feasibility Randomized Controlled Trial

**DOI:** 10.2196/48958

**Published:** 2023-12-22

**Authors:** Donghee N Lee, Rajani S Sadasivam, Elise M Stevens

**Affiliations:** 1 Department of Population and Quantitative Health Sciences Division of Preventive and Behavioral Medicine UMass Chan Medical School Worcester, MA United States; 2 Department of Population and Quantitative Health Sciences Division of Health Informatics and Implementation Science UMass Chan Medical School Worchester, MA United States

**Keywords:** mood, smoking cessation messages, computer-tailored health communication, innovation, smoking, cessation, digital intervention, effectiveness, text mining, adult, motivation

## Abstract

**Background:**

Computer-tailored health communication (CTHC), a widely used strategy to increase the effectiveness of smoking cessation interventions, is focused on selecting the best messages for an individual. More recently, CTHC interventions have been tested using contextual information such as participants’ current stress or location to adapt message selection. However, mood has not yet been used in CTCH interventions and may increase their effectiveness.

**Objective:**

This study aims to examine the association of mood and smoking cessation message effectiveness among adults who currently smoke cigarettes.

**Methods:**

In January 2022, we recruited a web-based convenience sample of adults who smoke cigarettes (N=615; mean age 41.13 y). Participants were randomized to 1 of 3 mood conditions (positive, negative, or neutral) and viewed pictures selected from the International Affective Picture System to induce an emotional state within the assigned condition. Participants then viewed smoking cessation messages with topics covering five themes: (1) financial costs or rewards, (2) health, (3) quality of life, (4) challenges of quitting, and (5) motivation or reasons to quit. Following each message, participants completed questions on 3 constructs: message receptivity, perceived relevance, and their motivation to quit. The process was repeated 30 times. We used 1-way ANOVA to estimate the association of the mood condition on these constructs, controlling for demographics, cigarettes per day, and motivation to quit measured during the pretest. We also estimated the association between mood and outcomes for each of the 5 smoking message theme categories.

**Results:**

There was an overall statistically significant effect of the mood condition on the motivation to quit outcome (*P*=.02) but not on the message receptivity (*P*=.16) and perceived relevance (*P*=.86) outcomes. Participants in the positive mood condition reported significantly greater motivation to quit compared with those in the negative mood condition (*P*=.005). Participants in the positive mood condition reported higher motivation to quit after viewing smoking cessation messages in the financial (*P*=.03), health (*P*=.01), quality of life (*P*=.04), and challenges of quitting (*P*=.03) theme categories. We also compared each mood condition and found that participants in the positive mood condition reported significantly greater motivation to quit after seeing messages in the financial (*P*=.01), health (*P*=.003), quality of life (*P*=.01), and challenges of quitting (*P*=.01) theme categories than those in the negative mood condition.

**Conclusions:**

Our findings suggest that considering mood may be important for future CTHC interventions. Because those in the positive mood state at the time of message exposure were more likely to have greater quitting motivations, smoking cessation CTHC interventions may consider strategies to help improve participants’ mood when delivering these messages. For those in neutral and negative mood states, focusing on certain message themes (health and motivation to quit) may be more effective than other message themes.

## Introduction

Smoking remains the leading cause of preventable disease and death in the United States [1]. Every 1 in 5 deaths can be attributed to smoking [2]. Smoking disparities are prevalent among marginalized populations (ie, racial or ethnic minoritized, lower socioeconomic status, and sexually minoritized groups), and it costs the United States more than US $225 billion annually [1,3]. To limit this health and economic burden, adult smoking cessation initiatives are needed [4], and most smokers want to quit [3] even though success rates in quitting are low [4]. Thus, innovative strategies are needed to help enable smokers to quit and stay quit.

One effective intervention for helping people who smoke quit is computer-tailored health communication (CTHC) [5-12]. CTHC is focused on selecting the best messages for an individual using computer algorithms. Traditional CTHC programs usually select messages based on participants’ demographic characteristics (eg, age and gender) or on key expert-identified variables (eg, a smoker’s readiness to quit) [13]. More advanced methods that use machine learning algorithms to select messages have also been developed [14,15]. CTHC messages can be delivered as a stand-alone program (eg, texting) or part of a complex intervention and has been integrated into several real-world health messaging programs [16,17]. Thus methods to increase the effectiveness of CTHC coupled with its potential for high reach can lead to interventions of high impact.

Recently, CTHC systems have incorporated contextual information about a participant—their current stress or location—as part of just-in-time interventions. This approach may be able to tailor smoking cessation messaging to another important smoker state (mood) to obtain better results. Mood, a temporary feeling state of recent affective responses (eg, feeling happy or depressed), influences one’s cognitions and behaviors [18-21], and as mood plays a vital role in customers’ purchasing decisions [22-24], many innovative companies (eg, Apple) have adopted mood tailoring within their advertisements [25-27]. Thus, it is possible that mood may be an important tailoring factor to increase the efficacy of smoking cessation digital interventions.

To begin to understand the association between mood and smoking cessation message effectiveness, we used crowdsourcing to examine how specific mood states (positive, negative, and neutral) impacted self-reported responses to smoking cessation messages about various topics (eg, financial costs or rewards, health, quality of life, challenges of quitting, and motivation or reasons to quit). The main research question of the study was as follows: According to a mood state, which messages increase motivation to quit, message receptivity, and perceived message relevance? The information gained from this study will help inform future digital smoking cessation interventions tailored to mood.

## Methods

### Participants

In January 2022, we recruited a convenience sample of adults who currently smoke cigarettes (N=615) from a web-based crowdsourcing survey platform, Prolific. Participants were eligible to participate if they were aged 18 years and older, currently smoked cigarettes (smoked at least 5 cigarettes a day and have smoked this amount for at least 1 year), and lived in the United States. We used prescreening measures on Prolific to determine participant eligibility.

### Procedures

Potential participants reviewed a brief description of the study on Prolific and were directed to Qualtrics, a web-based survey management system, for a 40-minute survey. After providing consent, participants completed questions about their tobacco use and motivation to quit smoking. Then, they were randomized to 1 of 3 mood conditions: positive, neutral, or negative. They viewed pictures within the assigned condition and indicated their mood afterward. Next, participants viewed a smoking cessation message and completed questions on message receptivity, perceived relevance, and motivation to quit smoking. The process was repeated 30 times. Finally, participants provided their sociodemographic characteristics. All participants saw the same pictures within their assigned mood condition, but all participants saw the same smoking cessation messages regardless of the random assignment. The pictures and messages were presented in random order.

### Ethical Considerations

The study was approved by the institutional review board at the UMass Chan Medical School (STUDY00000006) on November 5, 2021. Participants were provided with a consent form and gave consent electronically. Only those who consented were eligible for participation. We did not collect personally identifying information. Participants were compensated US $4.75 via Prolific per its policies.

### Stimuli

Each mood condition consisted of 30 pictures selected from the International Affective Picture System, a database of standardized pictures designed to induce a certain emotional state [28]. We used smoking cessation messages iteratively developed by a panel of experts and peer smokers from a large cohort study. Details are published elsewhere [13]. The messages had five theme categories: (1) financial costs or rewards (eg, “One pack of cigarettes per day for 10 years will cost you nearly US $25,000. How much are YOU spending on smoking?”); (2) health, such as physical ailments and health risks for oneself or others (eg, “Research shows that quitting smoking at any age can increase your life span by an average of 7 years.”); (3) quality of life, such as aesthetics and pleasant scents (eg, “Did you know that quitting smoking can give you a whiter smile, fresher breath, and clearer, younger looking skin?”); (4) challenges of quitting (eg, “People often smoke when they are stressed, to relax, after eating, and while driving. What triggers your smoking?”); and (5) motivation or reasons to quit (eg, “Are you worried about how smoking affects your family and friends? Try to avoid smoking around your loved ones.”). The top 6 highest-ranked messages from each theme category from a pilot study were selected for this study [13,15], resulting in a total of 30 messages (Table 1).

**Table 1:** Smoking cessation messages by theme categories^a^.

Theme and message			Ranking within theme
**Financial costs or rewards**			
	One pack of cigarettes per day for 10 years will cost you nearly US $25,000. How much are YOU spending on smoking?	1	
	Darcy, a former smoker, suggests saving all the money you spent on cigarettes as if you were buying them. Use it as a reward!	1	
	Advice from Andrea, a former smoker: Estimate how much you’ve spent on cigarettes daily, weekly, monthly, yearly. How much will it cost you over your lifetime?	1	
	If you smoke 1 pack of cigarettes/day, you are spending more than US $200 per month to smoke. What could you do with the money you’d save from quitting smoking?	2	
	The cost of smoking goes beyond the pack of cigarettes. Smokers have greater health care costs than nonsmokers because smoking causes many health problems.	3	
	Many health and life insurance companies charge lower premiums to nonsmokers.	4	
**Health**			
	Research shows that quitting smoking at any age can increase your life span by an average of 7 years.	1	
	Michael, a former smoker, thinks it’s important to quit because it helps extend your life with fewer health problems. It also saves money for other things.	1	
	COPD^b^ is the 4th leading cause of death in the United States. COPD is not fully reversible, but quitting smoking can help you breathe better and feel better.	2	
	What is your reason to quit? Valerie, a former smoker, said being physically away from her kids and the noise in her head to get away to smoke bothered her.	2	
	Long term risks of smoking include heart attacks and stroke, cancer, osteoporosis, and long-term disability.	3	
	Smoking can make breathing hard. After you quit you may breathe better and have more energy. Quitting also lowers your risk of getting cancer from smoking.	4	
**Quality of life**			
	Did you know that quitting smoking can give you a whiter smile, fresher breath and clearer, younger looking skin?	1	
	When you quit smoking, you will gain an improved sense of well-being. You can enjoy activities without feeling exhausted. It’s time to think about quitting.	2	
	The smell of smoke gets into your clothes, your car, your home, your hair, and your skin. No amount of air-freshener or perfume can fully mask this smell.	2	
	No matter how many years you have been smoking, quitting can increase your life span and give you a better quality of life.	3	
	Smoking depletes the skin’s natural glow and creates fine lines. Quitting smoking can help reverse the harm that smoking has done to your skin.	3	
	Quitting will have a positive impact on your physical ability and will help you perform better in your life. You can do this. Your doctor is ready to help.	3	
**Challenges of quitting**			
	People often smoke when they are stressed, to relax, after eating, and while driving. What triggers your smoking?	1	
	The worst withdrawal symptoms will occur in the first week after quitting, but by 1 month, most symptoms are gone.	2	
	Feelings of stress are normal when quitting smoking. You are not alone! Talk with your doctor or a friend about ways to reduce stress before your quit date.	3	
	Realize the first 48 hours after quitting is the most difficult time. Make a plan to handle it. It gets better!	4	
	There will be challenges to quitting, especially during the first few weeks. Make a list of things you can do, like exercise, to help with these challenges.	5	
	Did you know smoking can influence your mood? If you feel lonely or depressed while quitting, talk with your doctor. There is treatment to help.	6	
**Motivation to quit or reasons to quit**			
	Are you worried about how smoking affects your family and friends? Try to avoid smoking around your loved ones.	1	
	Tina, a young smoker, said it’s important for you to think about longevity. Many people want to see their children grow	1	
	Need another reason to quit smoking? Quitting may help you feel better about yourself and will help keep your children healthier.	2	
	Brandon, a current smoker, said that to get focused on quitting you should get psyched about a vacation, and the money you spend on cigs could go to that.	2	
	Make a list of why you want to quit smoking. Each day, use the list as a reminder of your reasons for wanting to quit.	3	
	Along with all the other health benefits of quitting, you will also notice improvement in the appearance of your hands and nails once you quit.	4	

^a^Adults who smoke cigarettes (N=615) were recruited from Prolific and saw 30 smoking cessation messages previously tested by experts and smokers from a large cohort study. Messages had 5 theme categories, and the top 6 highest-ranked messages from each theme category were selected for this study.

^b^COPD: chronic obstructive pulmonary disease.

### Measures

#### Demographics

Participants provided their age (in years), gender (collapsed to men, women, and nonbinary), race (collapsed to White and non-White), ethnicity (collapsed to not Hispanic or Latino and Hispanic or Latino), education (collapsed to high school or less, some college, and college graduate), marital status (collapsed to married and unmarried), self-perceived health status (treated as categorical), and financial stress (treated as categorical; Table 2).

**Table 2:** Baseline sociodemographic characteristics (n=567)^a^.

Characteristic			Value
Age (years), mean (SD)			41.13 (11.33)
**Gender, n (%)**			
	Men	262 (46.2)	
	Women	299 (52.7)	
	Nonbinary or other^b^	6 (0.11)	
**Race, n (%)^c^**			
	Asian	5 (0.9)	
	Black or African American	35 (6.2)	
	Native American or Alaska Native	7 (1.2)	
	Native Hawaiian or Pacific Islander	2 (0.4)	
	White	489 (86.4)	
	Not sure	2 (0.4)	
	Others^b^	26 (4.6)	
**Ethnicity, n (%)**			
	Hispanic or Latino	40 (7.1)	
	Not Hispanic or Latino	522 (92.1)	
	Do not know, not sure	5 (0.9)	
**Education, n (%)**			
	High school or less	124 (21.9)	
	Some college	241 (42.5)	
	College graduate	202 (35.6)	
**Marital status, n (%)**			
	Married	207 (36.5)	
	Divorced	75 (13.2)	
	Widowed	17 (3.0)	
	Separated	20 (3.5)	
	Never married	165 (29.1)	
	A member of an unmarried couple	83 (14.6)	
**Self-perceived health, n (%)**			
	Excellent	31 (5.5)	
	Very good	122 (21.5)	
	Good	243 (42.9)	
	Fair	153 (27.0)	
	Poor	18 (3.2)	
**Financial stress, n (%)**			
	Extremely difficult	74 (13.1)	
	Very difficult	68 (12.0)	
	Somewhat difficult	129 (22.8)	
	Slightly difficult	142 (25.0)	
	Not difficult at all	154 (27.2)	
Cigarettes smoked per day, n (%)			13.47 (8.2)
Pretest motivation to quit, n (%)			4.83 (2.6)

^a^Adults who smoke cigarettes (N=615) were recruited from Prolific. This table reports the characteristics of the analytic sample (n=567) with complete responses.

^b^Other include prefer not to answer, none of the above, and more than 1 racial category.

^c^A total of 22 participants selected more than 1 race category.

#### Cigarettes per Day

Participants were asked to report the average number of cigarettes smoked per day (range 0-30) [29].

#### Mood Conditions

Manipulation of the mood condition was confirmed using the Positive and Negative Affect Schedule (PANAS) self-report scale items assessing participants’ general mood state on a scale from 1 (not at all) to 5 (very much) [30]. After each of the first 3 mood induction images (ie, positive, negative, or neutral), participants were asked to rate their mood state on the PANAS scale. The PANAS scale contains 5 positive mood items (alert, inspired, determined, attentive, and active; Cronbach α=.88) and 5 negative mood items (upset, hostile, ashamed, nervous, and afraid; Cronbach α=.87). Items were summed by each picture and averaged across the mood conditions.

#### Motivation to Quit Smoking

Motivation to quit smoking was measured by using a single item from 1 (not at all motivated or confident) to 10 (very motivated or confident) to quit smoking. This question was used for both the pre- and posttest assessment [31].

#### Message Receptivity

Message receptivity was measured by adapting 10 items from the message receptivity scale. We asked the extent to which participants felt the message was appealing, spoke to them, said something important to them, convincing, would motivate persons to prevent smoking, confusing, promoted behaviors that are difficult, did not like the messages, and contradicted what they know about smoking on a scale from 1 (strongly disagree) to 5 (strongly agree). The last 4 items were reverse coded during analysis [32].

#### Perceived Relevance

Perceived relevance was measured by adapting 3 items from the perceived relevance scale. We asked the extent to which participants felt the message was relevant to their lives, grasped their attention, and said something important to them on a scale from 1 (strongly disagree) to 7 (strongly agree) [33].

### Statistical Analysis

We used SPSS (version 28.1; IBM Corp) for analysis. Among the total 615 participants, our analytic sample consisted of 567 participants who were successfully assigned to our experimental conditions. We ran 1-way ANOVA tests to estimate the associations between mood and outcomes (message receptivity, perceived relevance, and motivation to quit smoking). Next, we ran additional models to estimate the association between mood and outcomes for each of the 5 smoking message theme categories. Models controlled for sociodemographics, cigarettes per day, and motivation to quit smoking measured pretest. Additionally, a 1-tailed paired sample *t* test was performed to compare pre- and posttest motivation to quit smoking.

## Results

### Participant Characteristics

The mean age of the participants was 41.13 (SD 11.33) years. The majority of participants self-identified as women (299/567, 52.7%), White (489/567, 86.4%), and non–Hispanic or Latino (522/567, 92.1%); received some college degree (241/567, 42.5%); described their health as “good” (243/567, 42.9%); and described their financial life as not difficult at all (154/567, 27.2%). On average, most participants smoked 13.47 cigarettes per day and had an average of 4.83 (SD 2.65) scores of motivation or confidence to quit smoking (Table 2). In addition, participants’ motivation to quit smoking increased significantly from pretest (mean 4.82, SD 2.65) to posttest (mean 5.55, SD 2.53) assessment (t_610_=–9.550, *P*<.001).

### Mood Conditions

The manipulation of the mood condition was successful. Participants in the positive condition (mean 3.02, SD 0.97) reported higher average positive mood scores than those in the negative (mean 2.97, SD 0.96) or neutral (mean 2.88, SD 0.98) conditions. Participants in the negative condition (mean 1.75, SD 0.76) reported higher negative mood than those in the positive (mean 1.60, SD 0.76) or neutral (mean 1.58, SD 0.67) conditions.

### Association of Mood on Motivation to Quit, Message Receptivity, and Perceived Relevance: Overall and by Message Theme Categories

There was an overall statistically significant effect of the mood condition on the motivation to quit outcome (*P*=.02), but not on the message receptivity (*P*=.16) and perceived relevance outcomes (*P*=.86; Table 3). Further, participants in the positive mood condition reported significantly greater motivation to quit compared with those in the negative mood condition (*P*=.005), but not compared with those in the neutral condition (*P*=.06).

**Table 3:** Associations of mood conditions and outcomes^a^.

Measure and mood			Score, mean (SE)		*F* test (*df*)			*P* value	
**Message receptivity**						1.833 (2, 545)			.16
	Positive	3.69 (0.04)			(vs Negative)			.06	
	Negative	3.58 (0.04)			(vs Netural)			.41	
	Neutral	3.63 (0.04)			(vs positive)			.27	
**Perceived relevance**						0.156 (2, 545)			.86
	Positive	4.36 (0.10)			(vs Negative)			.59	
	Negative	4.29 (0.10)			(vs Neutral)			.89	
	Neutral	4.31 (0.10)			(vs Positive)			.69	
**Motivation to quit^b^**						4.028 (2, 545)			.02
	Positive^b^	5.86 (0.13)			(vs Negative)			.005	
	Negative^b^	5.36 (0.13)			(vs Neutral)			.35	
	Neutral	5.53 (0.12)			(vs Positive)			.06	

^a^Adults who smoke cigarettes (N=615) were recruited from Prolific and randomized to 1 of 3 mood conditions. One-way ANOVA tests were used to model the association between mood and message receptivity, perceived relevance, and motivation to quit. Models adjusted for covariates (cigarettes per day, pretest quitting motivation, age, gender, race, ethnicity, relationship status, self-perceived health, and financial stress). Pairwise comparisons of mood conditions were performed using the least significant difference when the overall ANOVA test was significant. The mean difference between the positive and negative mood conditions on the motivation to quit outcome was significant at α=.05.

^b^Statistical significance. This study was cross-sectional.

We compared the effects of the mood condition on the motivation to quit outcome for each of the 5 message theme categories: financial, health, quality of life, challenges of quitting, and motivation to quit (Table 4). We found an overall difference in the effects of the mood condition on the motivation to quit outcome in 4 of the 5 message theme categories: financial (*P*=.03), health (*P*=.01), quality of life (*P*=.04), and challenges of quitting (*P*=.03). However, the effect of the mood condition on the motivation to quit outcome was not statistically significant for motivation to quit themed messages (*P*=.07). Compared with those in the negative mood condition, participants in the positive mood condition reported significantly greater motivation to quit after seeing messages in the financial (*P*=.01), health (*P*=.003), quality of life (*P*=.01), and challenges of quitting (*P*=.01) theme categories, respectively. The overall association of the mood condition on message receptivity and perceived relevance was not statistically significant for any of the message theme categories (Table 4).

**Table 4:** Associations of mood conditions and outcomes by message theme categories^a^.

Measures, message theme, and mood			Mean (SE)	*F* test (*df*)		*P* value
**Message receptivity**						
	**Financial**				1.168 (2, 545)	.31
		Positive	3.74 (0.05)			
		Negative	3.64 (0.05)			
		Neutral	3.69 (0.05)			
	**Health**				2.648 (2, 545)	.07
		Positive	3.79 (0.05)			
		Negative	3.64 (0.05)			
		Neutral	3.72 (0.04)			
	**Quality of life**				2.690 (2, 545)	.07
		Positive	3.81 (0.05)			
		Negative	3.66 (0.05)			
		Neutral	3.75 (0.05)			
	**Challenges of quitting**				1.051 (2, 545)	.35
		Positive	3.50 (0.05)			
		Negative	3.42 (0.04)			
		Neutral	3.42 (0.04)			
	**Motivation to quit**				0.781 (2, 547)	.46
		Positive	3.62 (0.04)			
		Negative	3.54 (0.04)			
		Neutral	3.57 (0.04)			
**Perceived relevance**						
	**Financial**				0.361 (2, 545)	.70
		Positive	4.51 (0.12)			
		Negative	4.39 (0.12)			
		Neutral	4.40 (0.11)			
	**Health**				0.507 (2, 545)	.60
		Positive	4.58 (0.11)			
		Negative	4.43 (0.11)			
		Neutral	4.55 (0.11)			
	**Quality of life**				0.396 (2, 545)	.67
		Positive	4.52 (0.11)			
		Negative	4.42 (0.11)			
		Neutral	4.55 (0.11)			
	**Challenges of quitting**				0.265 (2, 545)	.77
		Positive	4.09 (0.11)			
		Negative	4.07 (0.11)			
		Neutral	3.99 (0.11)			
	**Motivation to quit**				0.125 (2, 545)	.88
		Positive	4.11 (0.10)			
		Negative	4.13 (0.10)			
		Neutral	4.06 (0.10)			
**Motivation to quit**						
	**Financial^b^**				3.506 (2, 545)	.03
		Positive^b^	5.98 (0.15)			
		Negative^b^	5.43 (0.15)			
		Neutral	5.59 (0.14)			
	**Health^b^**				4.409 (2, 545)	.01
		Positive^b^	6.21 (0.14)			
		Negative^b^	5.61 (0.14)			
		Neutral	5.83 (0.14)			
	**Quality of life^b^**				3.341 (2, 545)	.04
		Positive^b^	6.05 (0.15)			
		Negative^b^	5.52 (0.14)			
		Neutral	5.80 (0.14)			
	**Challenges of quitting^b^**				3.390 (2, 545)	.03
		Positive^b^	5.48 (0.13)			
		Negative^b^	5.01 (0.13)			
		Neutral	5.12 (0.13)			
	**Motivation to quit**				2.629 (2, 545)	.07
		Positive	5.59 (0.12)			
		Negative	5.24 (0.12)			
		Neutral	5.29 (0.11)			

^a^Adults who smoke cigarettes (N=615) were recruited from Prolific and randomized to 1 of 3 mood conditions. One-way ANOVA tests were used to model the association between mood and message receptivity, perceived relevance, and motivation to quit by message theme. Models adjusted for covariates (cigarettes per day, pretest quitting motivation, age, gender, race, ethnicity, relationship status, self-perceived health, and financial stress). Results were stratified by message theme. Pairwise comparisons of mood conditions were performed using the least significant difference when the overall ANOVA test was significant. The mean difference between the positive and negative mood conditions on the motivation to quit outcome was significant at α=.05 for the financial, health, quality of life, and challenges of quitting theme messages. Associations for conditions on message receptivity and perceived relevance were not statistically significant.

^b^Statistical significance. This study was cross-sectional.

## Discussion

### Principal Findings

This study formatively examined the effects of mood on smoking cessation message responses among adults who currently smoke cigarettes to inform the design of a mood-tailored smoking cessation CTHC intervention. Participants reported greater posttest motivation to quit compared with the pretest, which suggests that exposure to smoking cessation messages increased their motivation to quit smoking. Mood affected message responses, in that participants in the positive mood condition reported higher motivation to quit smoking than those in the negative mood conditions. Specifically, participants in the positive mood condition reported greater motivation to quit smoking after exposure to messages in most of the theme categories (ie, financial, health, quality of life, and challenges of quitting themes), except for messages in the motivation to quit theme category. We discuss implications for future CTHC design based on the results below.

The finding that participants in the positive mood condition were most likely to increase their motivation to quit may suggest that future smoking cessation interventions should use strategies to improve the mood of their participants. Prior studies have also noted the impact of positive mood on improving smoking cessation outcomes [34-37]. A prior study using a mood and stress management intervention found that participants who received treatment to boost their mood into a more positive state reported higher smoking cessation intervention retention rates than those without the mood treatment [34]. Other studies found an association between positive baseline emotions and higher smoking cessation intervention adherence and smoking abstinence, in contrast to negative baseline emotions that were associated with intervention dropouts and low smoking abstinence [35-37]. In a randomized controlled trial, text messages promoting positive feelings and encouragement increased long-term smoking cessation outcomes in digital smoking cessation intervention participants [38]. However, these studies did not specifically examine how the mood of the participants affected their response to different types of smoking cessation messages in the context of a mood-based CTHC development, which we report in this study.

In our study, participants in the positive mood condition reported a statistically significant increase in their motivation to quit following the financial, health, quality of life, and challenges of quitting themed messages. This finding may suggest that these messages should be delivered only when participants are in a positive mood state but not in the other (negative or neutral) mood states. However, considering that the magnitude of the increases in motivation may provide more granular information for the design of CTHC systems even for those in the neutral or negative states. For example, participants across the mood states reported the highest score in their motivation to quit following the health and quality of life themes. Comparatively, the scores for the challenges of quitting theme were relatively lower across the mood conditions. This may suggest that the health and quality of life themes may be appropriate for participants in all mood states, even though there were statistically significant differences in the mood conditions within these theme categories.

As noted, motivation to quit was not statistically different across the moods following the motivation to quit themed messages. This may either imply that these messages did not affect participants in any of the mood states or that messages may be effective across all mood states. In our study, the motivation to quit themed messages received higher mean scores for all 3 mood conditions than the challenges of quitting themed messages. However, mood did not affect message receptivity and perceived relevance of the messages in our study, although those in the positive mood state rated messages in all 5 themes higher than those in the negative or neutral mood states. One plausible explanation is that our participants saw each message once during this 1-time study, while repeated exposure to multiple messages may be necessary to influence message responses [39,40]. This finding provides information on message responses when there is only a single exposure. By using CTHC, researchers can tailor messages optimized to participants’ mood. Future research should adopt ecological momentary assessment tools to provide a repeated, real-time assessment of message responses based on participants’ mood to further investigate the effects of different themed messages.

Past research found differences in distinct dimensions of positive emotions (eg, hopefulness and pride) compared with general positive emotions (eg, overall happiness), and the specific role of pride in changing health behaviors [41]. Future analysis can examine how distinct mood types may interact with different message themes to influence message responses. Studies can also use qualitative methods to understand why individuals may respond differently to messages in motivation to quit theme compared with other message theme categories.

### Limitations

We have limitations and suggestions for future research. First, the majority of our sample identified as non-Hispanic White, although cigarette smoking is disproportionally prevalent among racial and ethnic minoritized individuals (non-Hispanic White: 13.3% vs non-Hispanic Black: 14.4% and American Indian or Alaska Native: 27.1%) [42]. Future studies should purposely oversample Black-identifying and American Indian individuals to examine the impact of mood on smoking cessation messages among this population to alleviate the cigarette use disparity. Relatedly, we used a web-based convenience sample. However, past tobacco-related studies found that using a convenience sample yielded comparable results with using a representative sample [43]. Our study used a cross-sectional design; thus it is possible that participant characteristics influenced our findings. Finally, the main outcomes were self-reported measures. Future studies can incorporate psychophysiological measures, such as eye-tracking and heart rate [44], to provide a more nuanced understanding of the impact of mood on responses to smoking cessation.

### Conclusions

Preliminary findings from our study indicate the importance of considering participants’ mood when designing CTHC interventions to support smoking cessation, specifically including short cessation messages as was tested in this study. Participants in positive mood were most likely to increase their motivation to quit following smoking cessation messages. As such, interventions may need to consider strategies to help improve participants’ mood when delivering smoking cessation messages. While those in the neutral and negative mood were less likely to increase their motivation to quit, certain message themes (health and motivation to quit) were more effective than other message themes. Future studies should investigate the real-time association of mood and message response by adopting ecological momentary assessment tools or behavioral sensors [41].

## Appendix

Multimedia Appendix 1 CONSORT checklist and participant flow diagram.

## Abbreviations

CTHC: Computer-tailored health communication
PANAS: Positive and Negative Affect Schedule

## Data Availability

The data sets generated or analyzed during this study are available from the corresponding author on reasonable request.
