# Supplementary material for: Developing Mood-Based Computer-Tailored Health Communication for Smoking Cessation: Feasibility Randomized Controlled Trial
Source: JMIR Form Res. 2023 Dec 22;7:e48958. doi: 10.2196/48958 (PMC10770788; doi:10.2196/48958)
Supplement: Multimedia Appendix 1 [file formative_v7i1e48958_app1.pdf]

CONSORT 2010 checklist of information to include when reporting a randomised trial*

Section/Topic	Item No	Checklist item	Reported on page No
**Title and abstract**			
	1a	Identification as a randomised trial in the title<br><br>We have identified the study as a randomized trial in the title: “Developing Mood-Based Computer-Tailored Health Communication for Smoking Cessation: Feasibility Randomized Controlled Trial”	
	1b	Structured summary of trial design, methods, results, and conclusions (for specific guidance see CONSORT for abstracts)<br><br>We have provided a structured abstract consisting of the trial background, objective, methods, results, and conclusions.	
**Introduction**			
Background and objectives	2a	Scientific background and explanation of rationale<br><br>Computer-tailored health communication (CTHC) is an effective intervention to help people who smoke to quit because it selects the best messages for an individual using computer algorithms. Delivering CTHC messages by tailored to contextual information about a participant (mood) as part of just-in-time interventions can help optimize the effectiveness of smoking cessation messaging.	
	2b	Specific objectives or hypotheses<br><br>The research question proposed: “According to a mood state, which messages increase motivation to quit, message receptivity, and perceived message relevance?”	
**Methods**			
Trial design	3a	Description of trial design (such as parallel, factorial) including allocation ratio<br><br>This study was a cross-sectional, randomized, parallel 3-armed design. Participants were randomly assigned to one of three mood arms in a one-time study.	
	3b	Important changes to methods after trial commencement (such as eligibility criteria), with reasons<br><br>No change from the standard CONSORT item.	

Section	Item	Checklist item
Participants	4a	Eligibility criteria for participants<br><br>Participants were eligible for the study if they currently smoked cigarettes (smoked at least 5 cigarettes a day and have smoked this amount for at least 1 year) and lived in the United States.
	4b	Settings and locations where the data were collected<br><br>We used Prolific, a web-based crowdsourcing survey platform, for data collection.
Interventions	5	The interventions for each group with sufficient details to allow replication, including how and when they were actually administered<br><br>Each group consisted of 30 mood-induction pictures from the International Affective Picture System selected for each of the mood condition (positive, negative, and neutral mood). Mood manipulation was checked using the PANAS self-report scale items. Then, participants were shown 30 smoking cessation messages in random order that were selected from a panel of health experts and former smokers in a prior study.
Outcomes	6a	Completely defined pre-specified primary and secondary outcome measures, including how and when they were assessed<br><br>The primary outcomes were motivation to quit, message receptivity, and perceived relevance. Motivation to quit outcomes was assessed pre-test and post-test using a single question item. Message receptivity was assessed post-test using 10 items from the message receptivity scale assessing the extent to which the message was appealing, spoke to them, said something important to them, convincing, would motivate persons to prevent smoking, confusing, promote behaviors that are difficult, did not like the messages, and contradicts what they know about smoking. Perceived relevance was assessed post-test using 3 items from the perceived relevance scale assessing the extent to which the message was relevant to their life, grasped their attention, and said something important.
	6b	Any changes to trial outcomes after the trial commenced, with reasons<br><br>No change from the standard CONSORT item.
Sample size	7a	How sample size was determined<br><br>This pilot study was conducted to get a more accurate estimate of the sample sizes needed for a larger, representative sample in the future. Therefore, we did not perform a power analysis for this study.
	7b	When applicable, explanation of any interim analyses and stopping guidelines

		Preliminary analysis was conducted using paired t-tests to assess pre-test and post-test differences in the primary outcome measure motivation to quit.	
Randomisation:			
Sequence generation	8a	Method used to generate the random allocation sequence<br><br>We used Qualtrics' pre-programmed, computer-generated feature to generate the random allocation sequence.	
	8b	Type of randomisation; details of any restriction (such as blocking and block size)<br><br>It was a simple randomization.	
Allocation concealment mechanism	9	Mechanism used to implement the random allocation sequence (such as sequentially numbered containers), describing any steps taken to conceal the sequence until interventions were assigned<br><br>Eligible participants were randomized in a 1:1:1 allocation to one of three arms using the Qualtrics' pre-programmed computer-generated random allocation feature.	
Implementation	10	Who generated the random allocation sequence, who enrolled participants, and who assigned participants to interventions<br><br>We used Qualtrics' pre-programmed, computer-generated feature to generate the random allocation. We used Prolific for participant recruitment and used Qualtrics' randomization feature to randomly assign the intervention arm.	
Blinding	11a	If done, who was blinded after assignment to interventions (for example, participants, care providers, those assessing outcomes) and how<br><br>All participants were blinded after the intervention assignment.	
	11b	If relevant, description of the similarity of interventions<br><br>All interventions (mood-induction pictures) were presented in the same format, size, and number (n=30), and in random order.	
Statistical methods	12a	Statistical methods used to compare groups for primary and secondary outcomes<br><br>We used 1-way ANOVA tests to estimate the association between the intervention and primary outcomes.	
	12b	Methods for additional analyses, such as subgroup analyses and adjusted analyses	

		When the overall ANOVA tests were statistically significant, we tested for the pairwise comparison of each arm on the primary outcomes controlling for cigarettes per day, pre-test quitting motivation, age, gender, race, ethnicity, relationship status, self-perceived health, and financial stress.	
**Results**			
Participant flow (a diagram is strongly recommended)	13a	For each group, the numbers of participants who were randomly assigned, received intended treatment, and were analysed for the primary outcome<br><br>See CONSORT-FLOW chart.	
	13b	For each group, losses and exclusions after randomisation, together with reasons<br><br>See CONSORT-FLOW chart.	
Recruitment	14a	Dates defining the periods of recruitment and follow-up<br><br>Participants were recruited for a one-time study in January 2022.	
	14b	Why the trial ended or was stopped<br><br>Data collection was completed because we met the accrual goal.	
Baseline data	15	A table showing baseline demographic and clinical characteristics for each group<br><br>Please refer to Table 1 for the baseline demographic characteristics of the total sample.	
Numbers analysed	16	For each group, number of participants (denominator) included in each analysis and whether the analysis was by original assigned groups<br><br>See CONSORT-FLOW chart.	
Outcomes and estimation	17a	For each primary and secondary outcome, results for each group, and the estimated effect size and its precision (such as 95% confidence interval)<br><br>We report the statistical significance	
	17b	For binary outcomes, presentation of both absolute and relative effect sizes is recommended<br><br>Binary outcomes were not included in analyses.	
Ancillary analyses	18	Results of any other analyses performed, including subgroup analyses and adjusted analyses, distinguishing	

		pre-specified from exploratory<br><br>Ancillary analyses were not performed.
Harms	19	All important harms or unintended effects in each group (for specific guidance see CONSORT for harms)<br><br>Participants were informed about the risks involving feeling psychological discomfort with answering questions about tobacco use and experiencing negative feelings for those assigned to the negative mood arm, and a possibility of breach of confidentiality.
**Discussion**		
Limitations	20	Trial limitations, addressing sources of potential bias, imprecision, and, if relevant, multiplicity of analyses<br><br>There are limitations involving sample, which consists of largely non-Hispanic White, and use of convenience sampling. Additionally, there is a limitation with cross-sectional design and self-report bias.
Generalisability	21	Generalisability (external validity, applicability) of the trial findings<br><br>Results of the trial may generalize to the population with similar characteristics (adults who smoke cigarettes and live in the U.S.).
Interpretation	22	Interpretation consistent with results, balancing benefits and harms, and considering other relevant evidence<br><br>We have reported interpretation of the study results informed by the literature in the Discussion section of the manuscript.
**Other information**		
Registration	23	Registration number and name of trial registry<br><br>N/A
Protocol	24	Where the full trial protocol can be accessed, if available<br><br>The trial protocol for the institution's institutional review board is available upon request (STUDY00000006 via UMass Chan Medical School).
Funding	25	Sources of funding and other support (such as supply of drugs), role of funders<br><br>The study was funded by iDAPT P50 Implementation Science Center in Cancer Control (MPI: RSS, EMS). Data analysis and manuscript preparation were additionally supported by R00DA046563(PI: EMS) via National

Institute on Drug Abuse and 2T32CA172009 (DNL) via National Institutes of Health.

Citation: Schulz KF, Altman DG, Moher D, for the CONSORT Group. CONSORT 2010 Statement: updated guidelines for reporting parallel group randomised trials. BMC Medicine. 2010;8:18. © 2010 Schulz et al. This is an Open Access article distributed under the terms of the Creative Commons Attribution License (http://creativecommons.org/licenses/by/2.0), which permits unrestricted use, distribution, and reproduction in any medium, provided the original work is properly cited.

*We strongly recommend reading this statement in conjunction with the CONSORT 2010 Explanation and Elaboration for important clarifications on all the items. If relevant, we also recommend reading CONSORT extensions for cluster randomised trials, non-inferiority and equivalence trials, non-pharmacological treatments, herbal interventions, and pragmatic trials. Additional extensions are forthcoming: for those and for up-to-date references relevant to this checklist, see www.consort-statement.org.

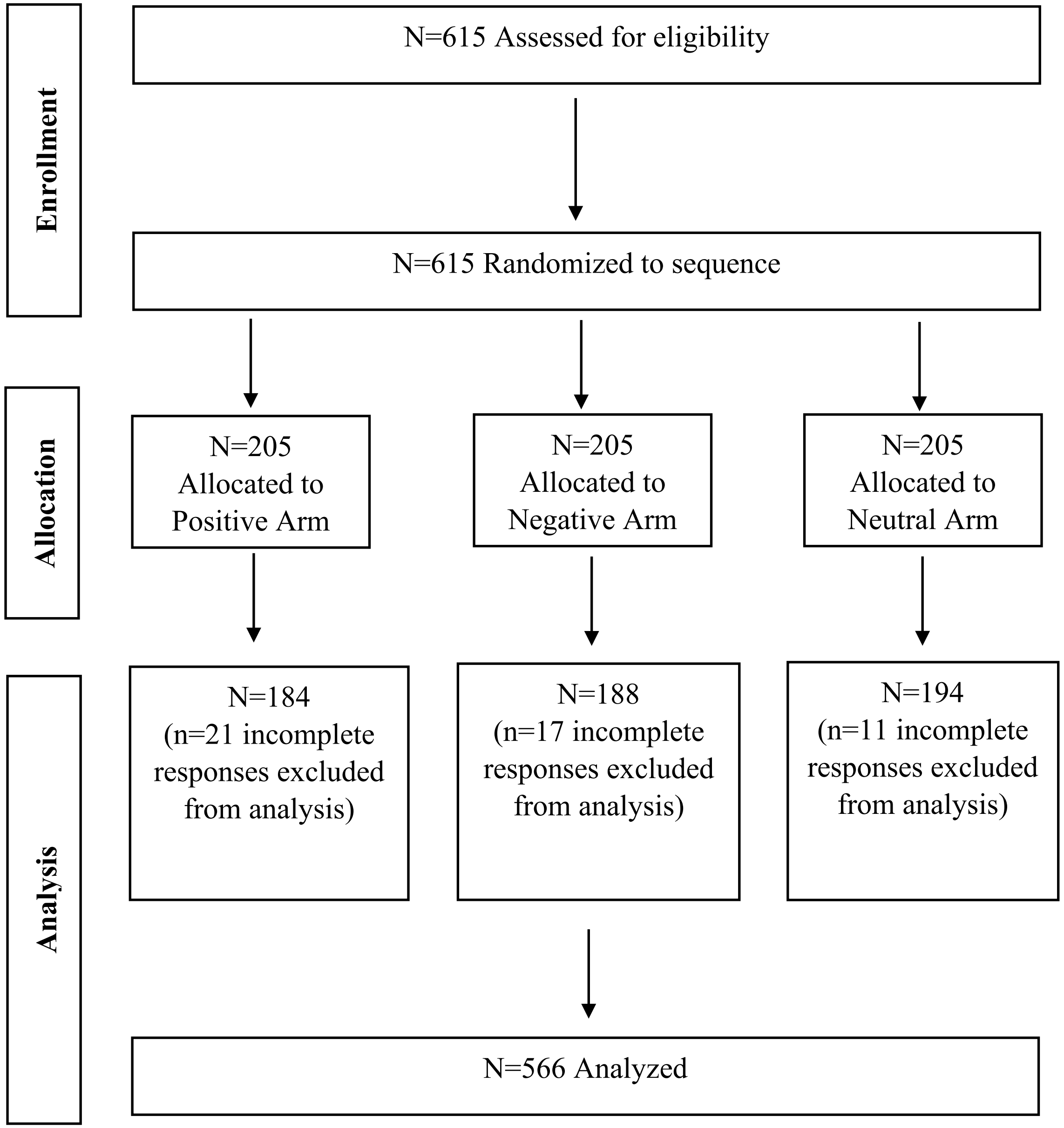

Flow diagram of the parallel 3-group randomized trial.
